# Supplementary material for: Integrative modelling of tumour DNA methylation quantifies the contribution of metabolism
Source: Nat Commun. 2016 Dec 14;7:13666. doi: 10.1038/ncomms13666 (PMC5171841; doi:10.1038/ncomms13666)
Supplement: Supplementary Information — Supplementary Figures 1-12, Supplementary Note 1 and Supplementary References. [file ncomms13666-s1.pdf]

Supplementary Figures

Supplementary Figure 1. Pan-cancer analysis of global and local DNA methylation variation

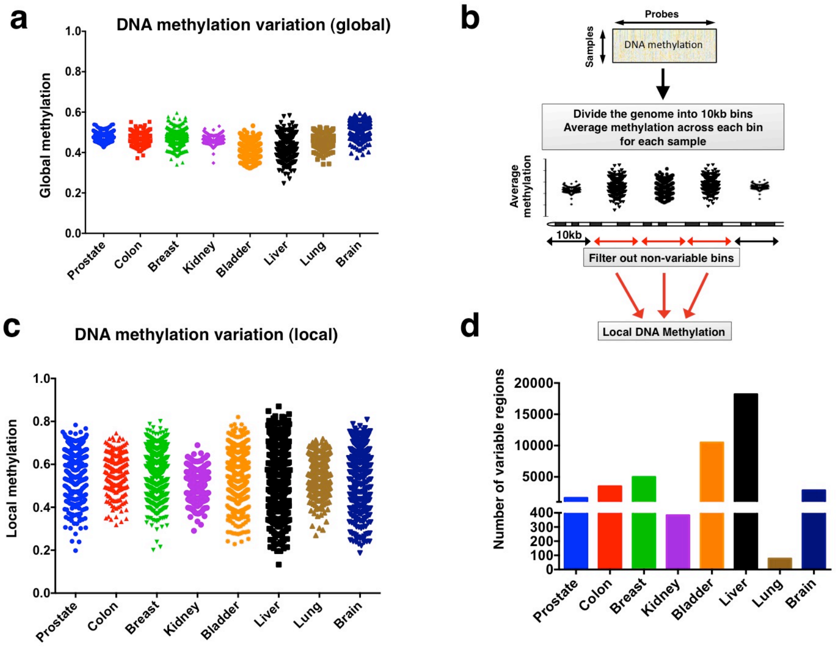

a) Variations in global DNA methylation are shown as measured by averaging the genome-wide value per sample. Values range between 0 and 1, with 1 indicating maximum methylation. Each point represents a unique tumor.

b) Schematic summarizing the approach used for dividing the genome into 10kb intervals and calculating local DNA methylations.

c) Variations in local DNA methylation. Each point represents a unique sample, and local DNA methylation is calculated as the average value across all selected 10kb regions.

d) Total number of 10kb bins across the genome with variable DNA methylation (sd > 0.2) is shown for each cancer type.

Supplementary Figure 2. Assessing models of local DNA methylation

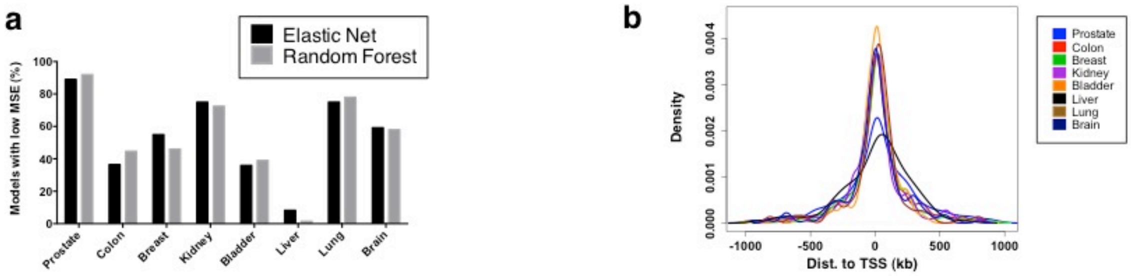

a) The y-axis shows the fraction of regions where DNA methylation was predicted with MSE smaller than 0.04 using the integrative models in each cancer.

b) Density plots resulting from positional annotation of regions where DNA methylation was most predictable (smallest MSEs) by the integrative models. Distance to nearest gene's transcription start site (TSS) is shown on the x-axis in kilobases.

Supplementary Figure 3. Comparison of our gene expression variables with popular gene families

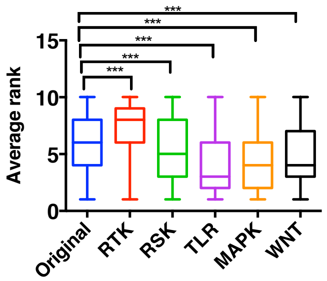

Comparison of original gene expression variables with 5 popular gene sets was considered: Receptor tyrosine kinases (RTK), Receptor serine kinases (RSK), Toll like receptors (TLR), MAPK signaling (MAPK) and WNT signaling (WNT) families. The y-axis shows the average rank of each gene expression category based on average variable importance score across all Random Forest models of local DNA methylation in brain cancer (Error bars show the minimum and maximum value in each group). Significance of p-values associated with the Mann-Whitney test between the ranks across all models is shown (***: <0.0001; see Methods).

Supplementary Figure 4. Results of modeling local DNA methylation

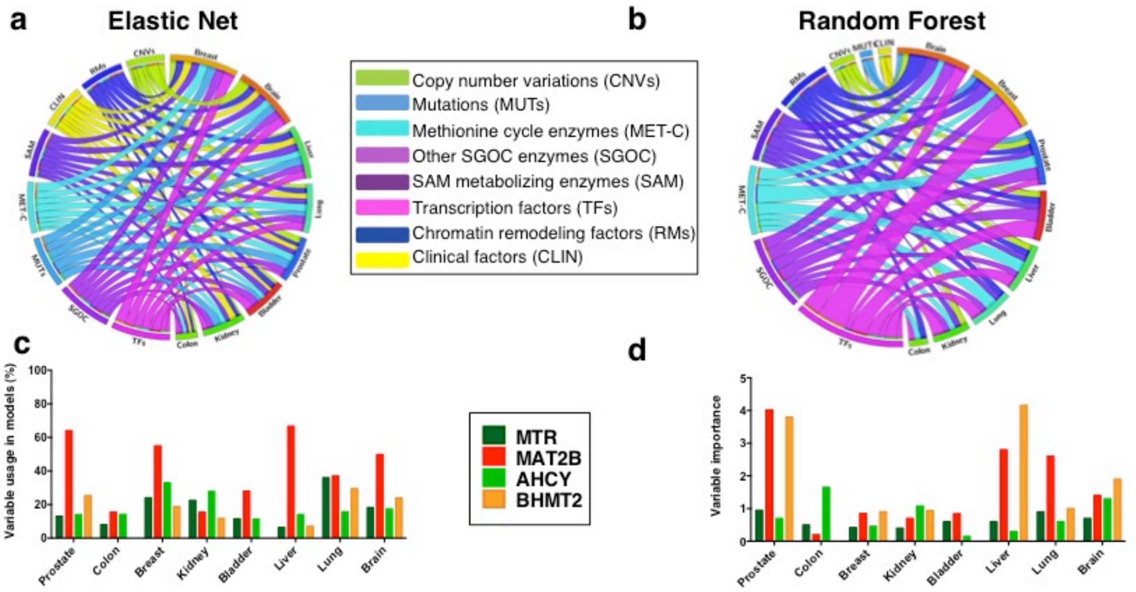

a) Contributions of variable classes to local DNA methylation are shown according to Elastic Net average variable usage (see Methods). The width of a given ribbon represents the relative value for the contribution of the corresponding variable class in the corresponding cancer type, with thicker ribbons showing higher relative contributions.

b) Contributions of variable classes to local DNA methylation are shown according to Random Forest average variable importance (see Methods).

c) The relative contributions of met cycle variables to local DNA methylation were calculated according to the Elastic Net integrative models with MSE<0.04. The y-axis shows the fraction of the 10kb regions wherein each variable was selected for prediction of DNA methylation (variable usage).

d) The relative contributions of met cycle variables to local DNA methylation were calculated according to the Random Forest integrative models with MSE<0.04. The y-axis shows average variable importance score across all models.

Supplementary Figure 5. Annotation and evaluation of metabolically regulated regions

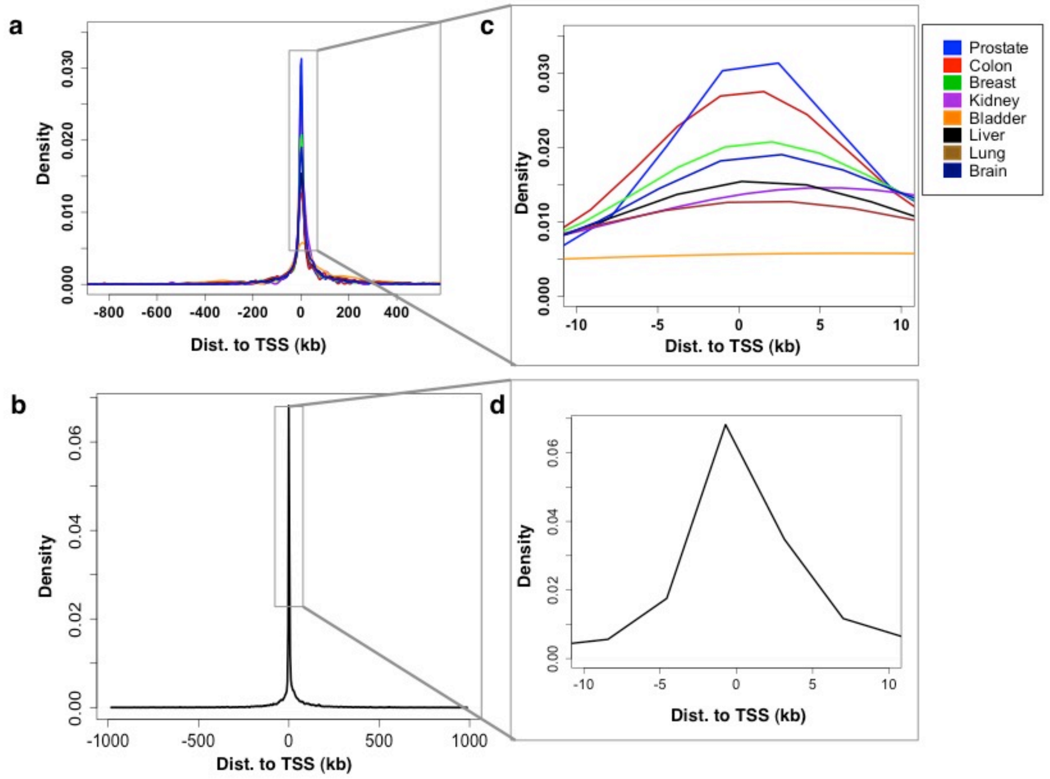

a) Density plots resulting from positional annotation of peaks identified in each cancer type by the genome-scanning algorithm described in Fig. 3 are depicted. Distance to nearest gene's TSS is shown on the x-axis in kilobases.

b) Density plots of the distribution around nearest gene's TSS for 10000 randomly selected probes along the Illumina Infinium HumanMethylation 450K BeadChip arrays are shown.

c) Zoomed-in view from part "a" to visualize the distribution of peaks immediately surrounding the TSS region.

d) Zoomed-in view from part "b" to visualize the distribution of probes immediately surrounding the TSS region.

Supplementary Figure 6. Test of specificity of the metabolically regulated regions for correlation with met cycle expression

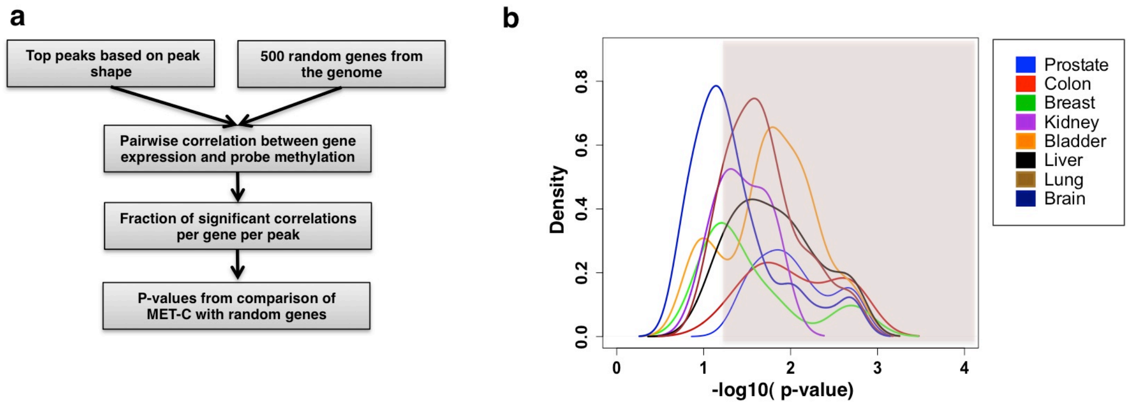

a) Diagram describing the method used for testing specificity of correlation peaks for the met cycle genes vs. random genes. A p-value is calculated for each peak by comparison to 500 random genes (see Methods).

b) Density plot of the distribution of randomization p-values for all peaks. Shaded area shows significant p-values (<0.05) indicating peaks that were specifically and non-randomly correlated with the met cycle genes.

Supplementary Figure 7. Functional annotation of metabolically regulated regions of the epigenome

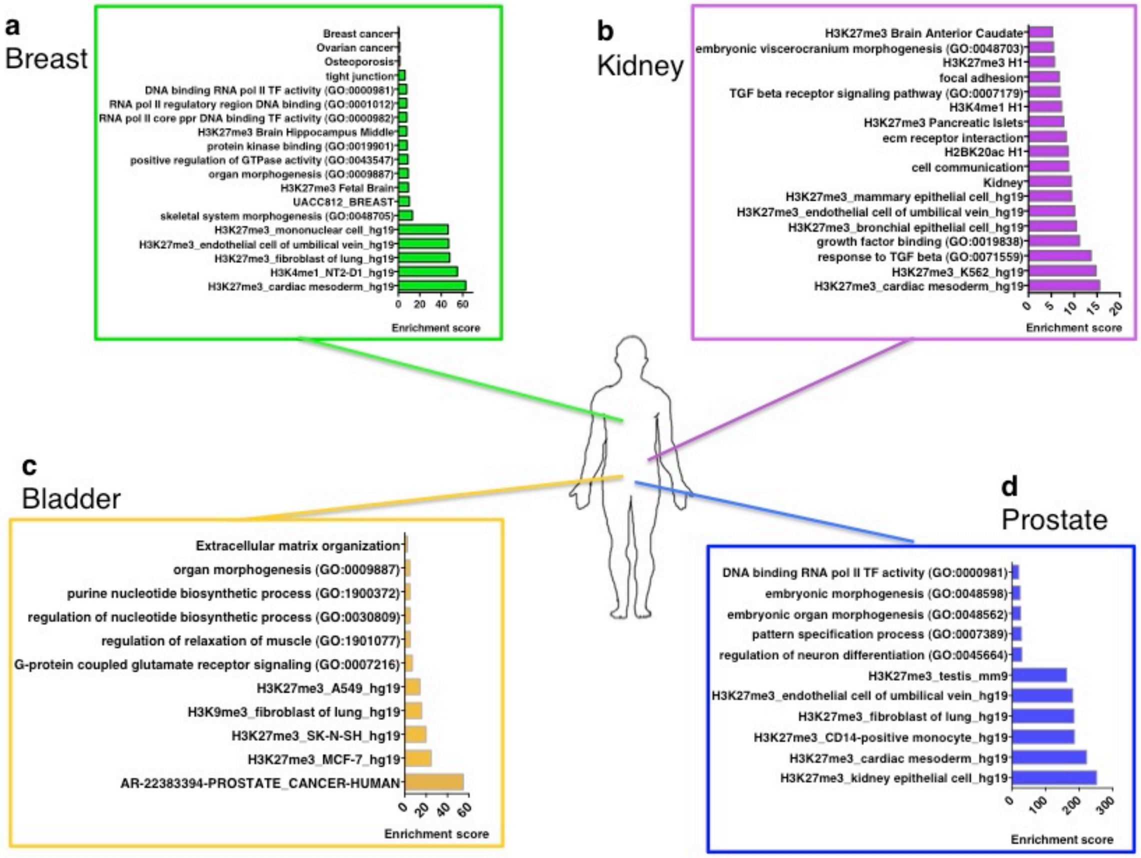

a-d) Pathway enrichment analyses results in cancers of breast, kidney, bladder, and prostate. Functional annotation analyses were performed on lists of genes located within peaks of correlation between met cycle and DNA methylation in corresponding cancers.

Supplementary Figure 8. Modeling DNA methylation at cancer gene promoters and gene bodies

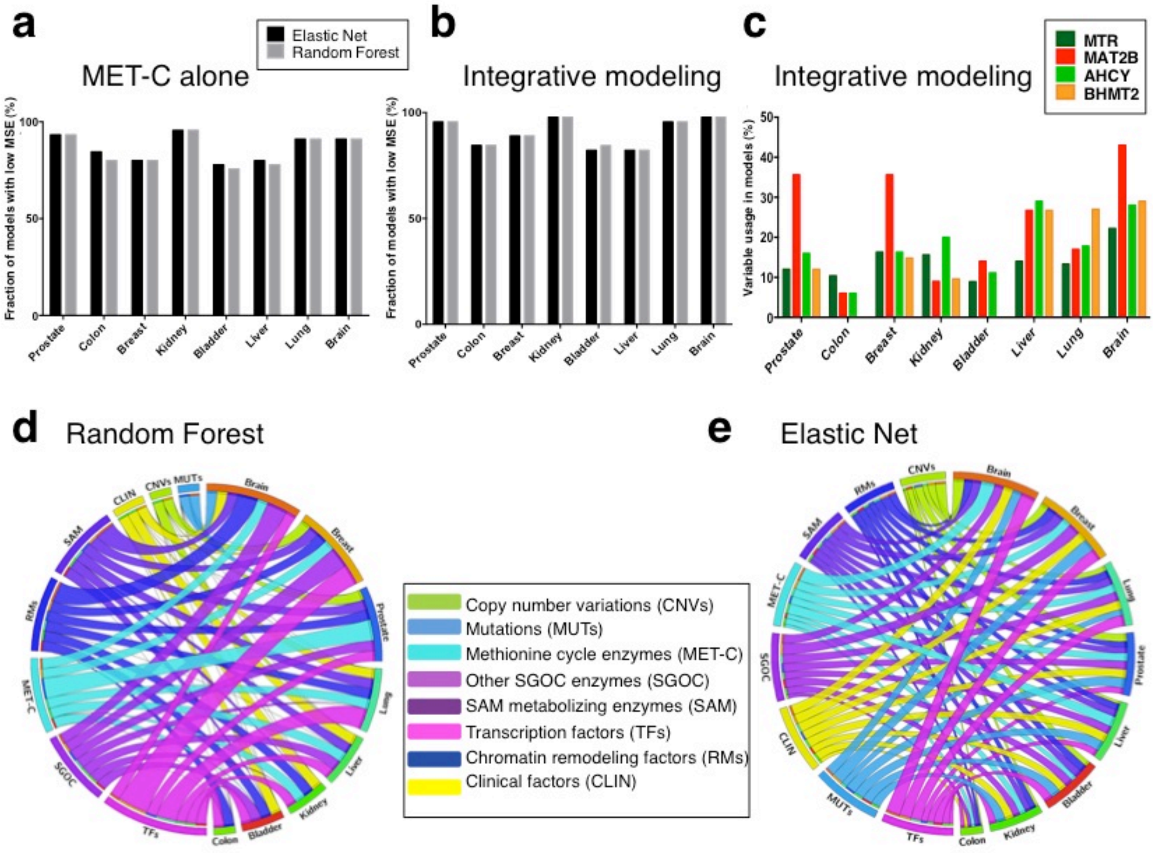

a) Fraction of cancer genes that were predictable by met cycle genes alone with test set prediction error (MSE) of 0.01 or smaller.

b) Fraction of genes that were predictable by the integrative models with test set prediction error (MSE) of 0.01 or smaller.

c) Fraction of Elastic Net models in which the met cycle variables was selected by the integrative approach in each cancer type.

d) Contribution of variable classes to cancer gene DNA methylation according to Random Forest average variable importance of each class (see Methods). The width of a given ribbon represents the relative value for the contribution of the corresponding

variable class in the corresponding cancer type, with thicker ribbons showing higher relative contributions.

e) Contribution of variable classes to cancer gene DNA methylation according to Elastic Net average variable usage of each class (see Methods).

Supplementary Figure 9. Evaluation of modeling performance using randomized responses

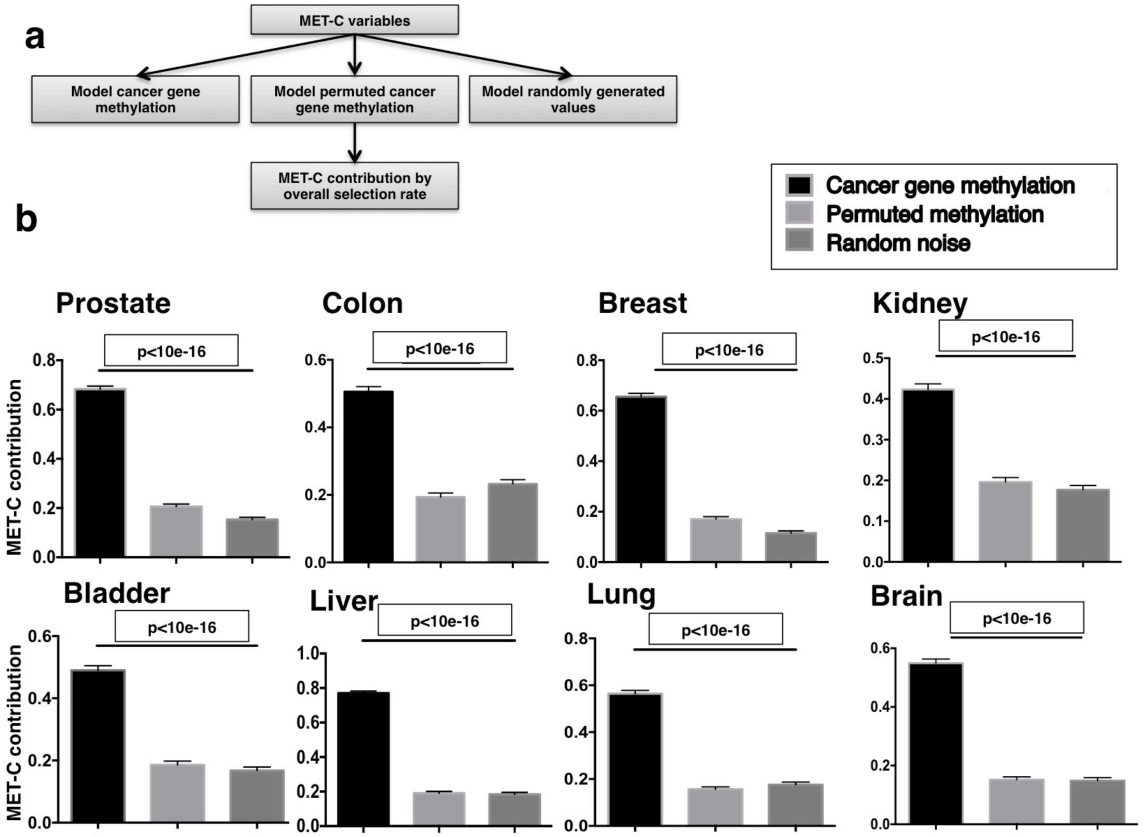

a) Diagram summarizing the approach used for testing the reliability of models by comparing cancer gene methylation values with randomized responses.

b) Average contribution of met cycle variables to prediction of cancer gene methylation vs. permutated methylation values and randomly generated numbers (see Methods). The y-axis shows the fraction of Elastic Net models wherein met cycle variables were selected. Kolmogorov-Smirnov non-parametric p-values were calculated between the variable usage values obtained using the original methylations vs. permutated or random responses separately. (Significant p-values (<10e-16) were also obtained by comparing the Random Forest variable importance scores across the models in all cases (not shown). Error bars show the standard error of mean (SEM) in each category)

Supplementary Figure 10. Evaluation of modeling performance using randomized predictors

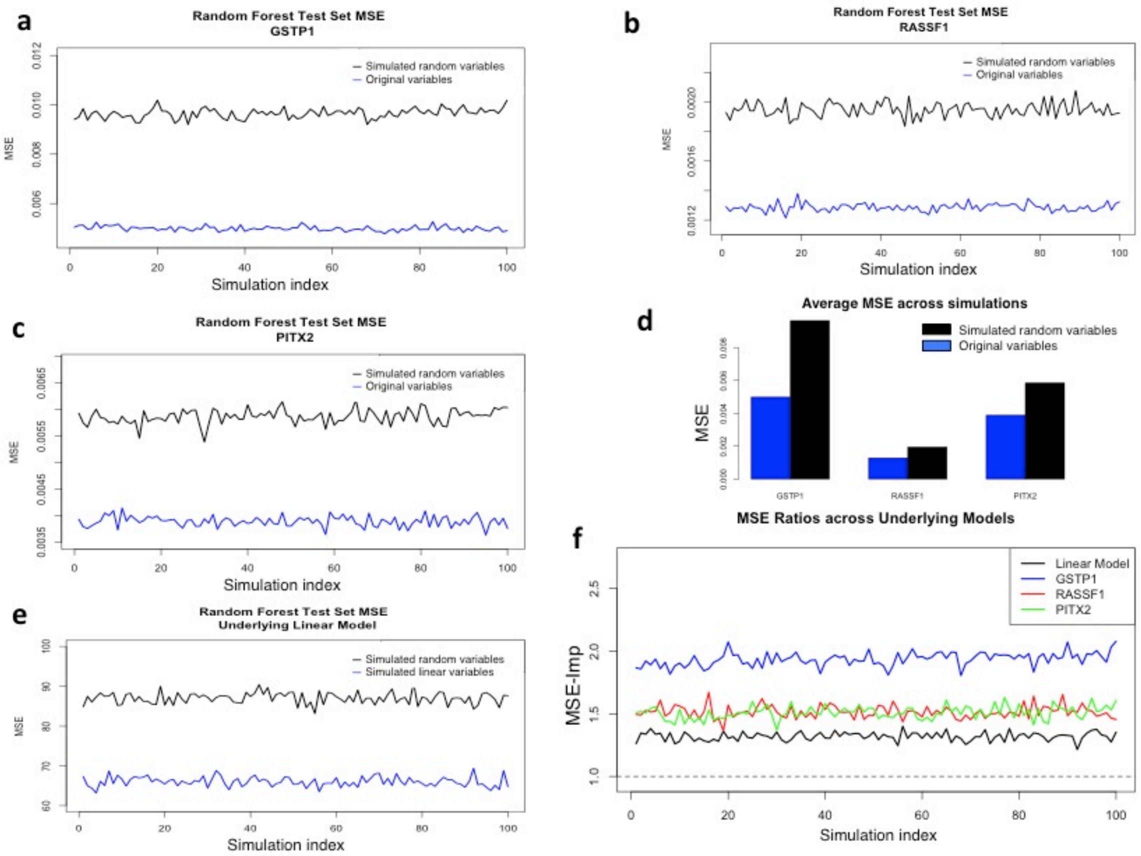

a) Comparison of glutathione S-transferase pi 1 (GSTP1) methylation prediction error by the original variables vs. random simulated variable sets of the same dimensions in prostate cancer (see Methods).

b) Comparison of RAS association domain family member-1 (RASSF1) methylation prediction error by the original variables vs. random simulated variable sets of the same dimensions in prostate cancer.

c) Comparison of paired-like homeodomain transcription factor 2 (PITX2) methylation prediction error by the original variables vs. random simulated variable sets of the same dimensions in prostate cancer.

d) Average MSE across 100 simulations of random predictors is shown for each of the responses.

e) Comparison of prediction errors for a simulated response by variables linearly related to the response vs. random variable set of the same dimensions.

f) Improvement of predictions by original variables vs. random variable (MSE-Imp= MSE-rand / MSE-orig) is plotted for the three example responses from our original dataset and also a simulated linearly-related dataset (see Methods). (MSE-rand= average MSE calculated using the randomly simulated variables, MSE-orig= average MSE calculated using the original variables)

Supplementary Figure 11. Summary of predictive modeling of DNA methylation at cancer gene loci

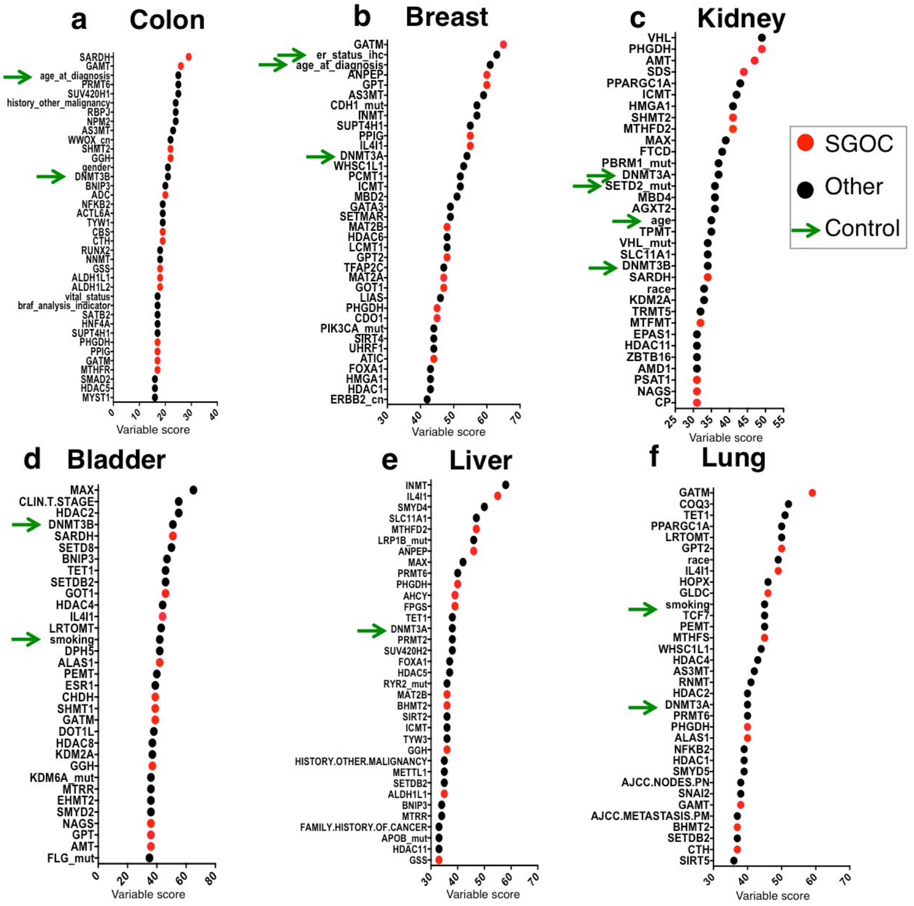

a-f) Variables that were most predictive of cancer gene methylation on average (top 15%) are listed and ranked in order of increasing contribution (variable score= percent variable usage by Elastic Net averaged across all models of cancer gene body and promoter methylation). Variables in the serine, glycine, one-carbon (SGOC) network (including the met cycle genes and other SGOC genes) are shown in red and all other variables are shown in black. Green arrows point to previously published factors associated with variations in DNA methylation in each cancer type (positive controls). (Variable names: official gene symbols are used to show gene expression variables (including "Methionine cycle enzymes", "Other SGOC enzymes", "Transcription Factors", "Chromatin

Remodelers”, and “ SAM-metabolizing enzymes”), while “_mut” and “_cn” suffixes following gene symbols denote “Mutations” and “Copy Number Variations”, respectively. For “Clinical factors”, variable names match the descriptors used in the TCGA clinical data files).

Supplementary Figure 12. Independent analyses of survival in TCGA cases by cBioPortal and PRECOG

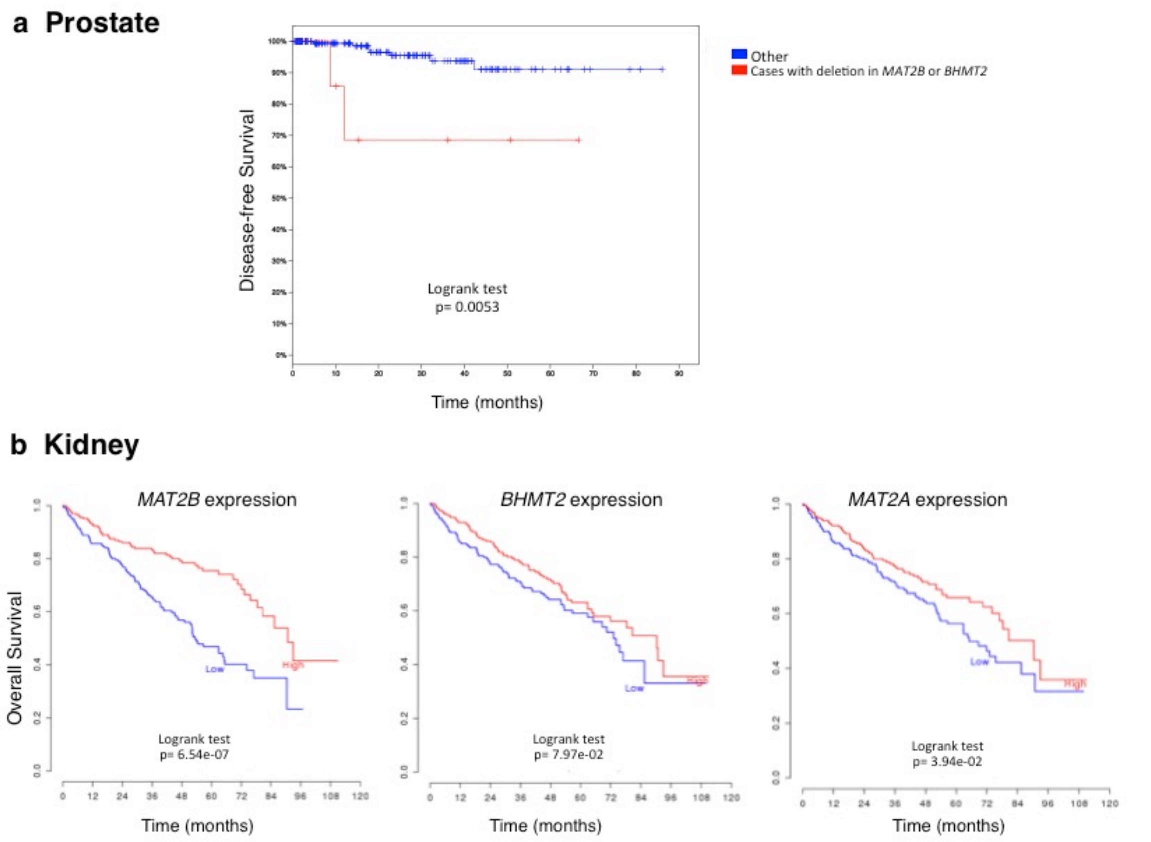

a) Comparison of disease-free survival between patients with deep deletions in *MAT2B* or *BHMT2* genes and other patients in the TCGA prostate cancer cohort. The plot and the log-rank test p-value were adopted from the cBioPortal.

b) Comparison of overall survival between TCGA kidney cancer patient groups exhibiting high expression vs. low-expression of the met cycle genes. Plots and log-rank test p-values were adopted from Prediction of Clinical Outcome from Genomic profiles (PRECOG). (MAT2B= methionine-adenosyltransferase 2B, MAT2A= methionine-adenosyltransferase 2A, BHMT2= betaine-homocysteine S-methyltransferase 2)

Supplementary Note 1

We considered an analysis of a large set of DNA methylation arrays from the TCGA that were collected and processed according to a standardized procedure that results in an estimate of the relative amount of DNA methylation at each oligonucleotide probe (the beta-value). This value ranges from 0 to 1 with 1 indicating that each allele is completely methylated[1]. Arrays were used over bisulfite sequencing because of the higher availability of these data in a standardized format allowing for an integrative analysis. We considered several tumor types with large sample sizes where both RNA-seq and DNA methylation data was available on each tumor (breast invasive carcinoma (BRCA), colon adenocarcinoma (COAD), lung adenocarcinoma (LUAD), liver hepatocellular carcinoma (LIHC), brain lower grade glioma (LGG), bladder urothelial carcinoma (BLCA), kidney renal clear cell carcinoma (KIRC), and prostate adenocarcinoma (PRAD).

Upon analysis of global DNA methylation levels (average per tumor), we observed that variation in global methylation across tumors from the same cancer type is higher than the between-cancer-type variation (between-cancer-type sum of squares (SS)= 44%, within-cancer-type SS= 56%) (Supplementary Fig. 1a). This differs from what is typically seen in normal tissues where between-tissue type variability in DNA methylation exceeds within-tissue type variability by an order of magnitude[2,3]. Thus, our results confirm increased inter-individual variation in DNA methylation among tumors from the same tissue of origin, consistent with methylation hypervariability in cancer[4]. It is important to note however, that due to the nature of the current TCGA data (one sample per tumor), we were unable to further parse this inter-individual variation to

distinguish between variations caused by differences between individual patients *vs*. differences between clonal populations of cells within a given tumor.

Since the biological function of DNA methylation occurs at specific regions of genomic DNA, we considered a local analysis of DNA methylation. We partitioned the genome into 10 kilobase (kb) regions and calculated average methylation in each region (Supplementary Fig. 1b; Methods). Notably, a previous study showed that DNA methylation at genomic regions with high inter-individual variation is more likely to be associated with expression of nearby genes, suggesting that variable regions are enriched for functionally active DNA methylation[5]. We therefore focused only on regions with standard deviation (sd) of 0.2 or higher for the subsequent integrative analyses (Supplementary Fig. 1c). The number of such regions differed substantially among cancer types, with liver and bladder cancers exhibiting the largest number of variable DNA methylation regions (Supplementary Fig. 1d).

Supplementary References

1. Cancer Genome Atlas, N. Comprehensive molecular portraits of human breast tumours. *Nature* 490, 61-70 (2012).
2. Ziller, M.J., *et al*. Charting a dynamic DNA methylation landscape of the human genome. *Nature* 500, 477-481 (2013).
3. Lokk, K., *et al*. DNA methylome profiling of human tissues identifies global and tissue-specific methylation patterns. *Genome biology* 15, r54 (2014).
4. Hansen, K.D., *et al*. Increased methylation variation in epigenetic domains across cancer types. *Nature genetics* 43, 768-775 (2011).
5. Gutierrez-Arcelus, M., *et al*. Passive and active DNA methylation and the interplay with genetic variation in gene regulation. *eLife* 2, e00523 (2013).
